# Association of Bilateral Salpingo-Oophorectomy Before Menopause Onset With Medial Temporal Lobe Neurodegeneration

**DOI:** 10.1001/jamaneurol.2018.3057

**Published:** 2018-10-15

**Authors:** Burcu Zeydan, Nirubol Tosakulwong, Christopher G. Schwarz, Matthew L. Senjem, Jeffrey L. Gunter, Robert I. Reid, Liliana Gazzuola Rocca, Timothy G. Lesnick, Carin Y. Smith, Kent R. Bailey, Val J. Lowe, Rosebud O. Roberts, Clifford R. Jack, Ronald C. Petersen, Virginia M. Miller, Michelle M. Mielke, Walter A. Rocca, Kejal Kantarci

**Affiliations:** 1 Department of Radiology, Mayo Clinic, Rochester, Minnesota; 2 Department of Neurology, Mayo Clinic, Rochester, Minnesota; 3 Division of Biomedical Statistics and Informatics, Department of Health Sciences Research, Mayo Clinic, Rochester, Minnesota; 4 Department of Information Technology, Mayo Clinic, Rochester, Minnesota; 5 Division of Epidemiology, Department of Health Sciences Research, Mayo Clinic, Rochester, Minnesota; 6 Department of Physiology and Biomedical Engineering, Mayo Clinic, Rochester, Minnesota; 7 Department of Surgery, Mayo Clinic, Rochester, Minnesota

## Abstract

**Importance:**

There is an increased risk of cognitive impairment or dementia in women who undergo bilateral salpingo-oophorectomy (BSO) before menopause. However, data are lacking on the association of BSO before menopause with imaging biomarkers that indicate medial temporal lobe neurodegeneration and Alzheimer disease pathophysiology.

**Objective:**

To investigate medial temporal lobe structure, white matter lesion load, and β-amyloid deposition in women who underwent BSO before age 50 years and before reaching natural menopause.

**Design, Setting, and Participants:**

This nested case-control study of women in the population-based Mayo Clinic Cohort Study of Oophorectomy and Aging-2 (MOA-2) and in the Mayo Clinic Study of Aging (MCSA) in Olmsted County, Minnesota, included women who underwent BSO from 1988 through 2007 and a control group from the intersection of the 2 cohorts. Women who underwent BSO and control participants who underwent a neuropsychological evaluation, magnetic resonance imaging (MRI), and Pittsburgh compound B positron emission tomography (PiB-PET) were included in the analysis. Data analysis was performed from November 2017 to August 2018.

**Exposure:**

Bilateral salpingo-oophorectomy in premenopausal women who were younger than 50 years.

**Main Outcomes and Measures:**

Cortical β-amyloid deposition on PiB-PET scan was calculated using the standard uptake value ratio. White matter hyperintensity volume and biomarkers for medial temporal lobe neurodegeneration (eg, amygdala volume, hippocampal volume, and parahippocampal-entorhinal cortical thickness) on structural MRI and entorhinal white matter fractional anisotropy on diffusion tensor MRI were also measured.

**Results:**

Forty-one women who underwent BSO and 49 control participants were recruited. One woman was excluded from the BSO group after diagnosis of an ovarian malignant condition, and 6 women were excluded from the control group after undergoing BSO after enrollment. Twenty control participants and 23 women who had undergone BSO completed all examinations. The median (interquartile range [IQR]) age at imaging was 65 (62-68) years in the BSO group and 63 (60-66) years in the control group. Amygdala volume was smaller in the BSO group (median [IQR], 1.74 [1.59-1.91] cm^3^) than the control group (2.15 [2.05-2.37] cm^3^; *P* < .001). The parahippocampal-entorhinal cortex was thinner in the BSO group (median [IQR], 3.91 [3.64-4.00] mm) than the control group (3.97 [3.89-4.28] mm; *P* = .046). Entorhinal white matter fractional anisotropy was lower in the BSO group (median [IQR], 0.19 [0.18-0.22]) than the control group (0.22 [0.20-0.23]; *P* = .03). Women were treated with estrogen in both groups (BSO, n = 22 of 23 [96%]; control, n = 10 of 19 [53%]). Global cognitive status test results did not differ between the groups.

**Conclusions and Relevance:**

Abrupt hormonal changes associated with BSO in premenopausal women may lead to medial temporal lobe structural abnormalities later in life. Longitudinal evaluation is needed to determine whether cognitive decline follows.

## Key Points

**Question:**

Do women who underwent bilateral salpingo-oophorectomy before menopause show greater medial temporal lobe structural changes, β-amyloid accumulation, and white matter lesion load on neuroimaging later in life compared with a control group?

**Findings:**

In this case-control study, women with bilateral salpingo-oophorectomy before menopause had smaller amygdala volumes, thinner parahippocampal-entorhinal cortices, and lower entorhinal white matter fractional anisotropy values compared with control participants.

**Meaning:**

Abrupt hormonal changes associated with bilateral salpingo-oophorectomy in premenopausal women may lead to medial temporal lobe structural abnormalities later in life; because alterations in structural imaging biomarkers of the medial temporal lobe neurodegeneration may precede clinical symptoms of dementia, longitudinal follow-up of this cohort with cognitive testing is necessary.

## Teaser

This case-control study combines portions of 2 existing cohorts to investigate medial temporal lobe structure, white matter lesion load, and β-amyloid deposition in older women who underwent bilateral salpingo-oophorectomy before age 50 years and before reaching natural menopause, compared with a group of female control participants.

## Introduction

Women who undergo bilateral salpingo-oophorectomy (BSO) before the onset of menopause have an accelerated accumulation of multimorbidity, with an increased risk of aging-associated neurological diseases, including dementia.^1,2,3^ Furthermore, surgical menopause at an early age was associated with Alzheimer disease (AD) pathology at autopsy.^4^ Because imaging biomarkers associated with cognitive impairment and dementia precede the clinical symptoms, and may provide insight into the underlying causative mechanisms of cognitive impairment and dementia later in life, we investigated β-amyloid deposition (primary outcome), magnetic resonance imaging (MRI)–based biomarkers of medial temporal lobe neurodegeneration, and white matter hyperintensity volume in women who underwent BSO before age 50 years and before reaching natural menopause.

## Methods

**Figure 1.**
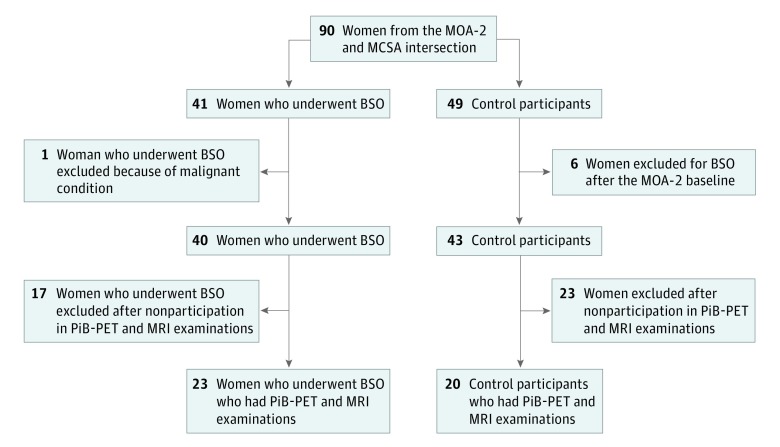
Study Sample BSO indicates bilateral salpingo-oophorectomy; MCSA, Mayo Clinic Study of Aging; MOA-2, Mayo Clinic Cohort Study of Oophorectomy and Aging-2; MRI, magnetic resonance imaging; PET, positron emission tomography; PiB, Pittsburgh compound B.

The Mayo Clinic Cohort Study of Oophorectomy and Aging-2 (MOA-2) is a population-based cohort study that includes women who underwent BSO before age 50 years and before reaching natural menopause from 1988 through 2007 and an age-matched control group who had not undergone bilateral oophorectomy before age 50 years.^5^ The Mayo Clinic Study of Aging (MCSA) is another population-based cohort study, which includes participants with normal cognitive aging, mild cognitive impairment, and dementia.^6^ All MCSA participants are invited to undergo MRI and Pittsburgh compound B (PiB) positron emission tomography (PET). Both study cohorts are representative of the geographically defined population of Olmsted County, Minnesota. In the current study, women with BSO and control-participant women from the MOA-2 cohort who later were enrolled in the MCSA and underwent a neuropsychological evaluation, MRI, and PiB-PET scan were included (Figure 1). This study was approved by the Mayo Clinic institutional review board, and written informed consent was obtained from all participants.

Global cognitive status was assessed using the short test of mental status, and combining 9 tests into a global cognitive status score.^6^ The MRI studies were performed at 3-T (GE Healthcare). Previously described and validated MRI analysis methods were used.^7,8^ Hippocampal and amygdala volumes were adjusted for total intracranial volume. Parahippocampal-entorhinal cortical thickness was measured using FreeSurfer version 5.3 (Athinoula A. Martinos Center for Biomedical Imaging at Massachusetts General Hospital).^9^ A semi-automated segmentation^10^ of fluid-attenuated inversion recovery (FLAIR) images was used for white matter hyperintensity volume quantification and adjusted for total intracranial volume. Cerebral infarcts were also evaluated. Previously described and validated methods were used to process diffusion tensor imaging scans.^11^ Entorhinal white matter fractional anisotropy, which includes the perforant pathway, was quantified using the Johns Hopkins University atlas.^12^ A PET and computed tomography scanner with 3-dimensional mode (GE Healthcare) was used for PET imaging, and image analysis was performed using an automated image processing pipeline.^8^ Cortical β-amyloid deposition on PiB-PET scan was calculated using standard uptake value ratio.

### Statistical Analysis

Wilcoxon rank sum tests, rank regression tests adjusted for total intracranial volume, and Fisher exact tests were used for comparisons of the BSO and control groups. Correlations between imaging biomarkers and global cognitive status scores were assessed using Spearman correlations, and the *P* values were adjusted for multiple comparisons using the false-discovery rate. Statistical significance was considered at the 2-sided α level of .05.

## Results

A total of 43 women fulfilled the inclusion criteria, including 23 who had undergone BSO and 20 control participants. The median age at oophorectomy in the BSO group was 46 (interquartile range [IQR], 45-48) years. Age at imaging and frequency of *APOE* ε4 carriers did not differ between the groups. Among the 23 women who underwent BSO with hysterectomy, 22 (96%) took unopposed estrogen for a median duration of 10 (IQR, 5-13) years after surgery. Oral conjugated equine estrogen was the most common type used (n = 15 of 22 [68%]), typically at a dosage of 0.625 mg/d. Among the 20 control-participant women, 19 (95%) reached menopause during the study follow-up, 17 (85%) had natural menopause, and 10 of 19 (53%) took estrogen for a median duration of 10 (IQR, 6-16) years. The most common type used was oral conjugated equine estrogen at a dosage of 0.625 mg/d (n = 9 of 10 [90%]) with progestin (oral medroxyprogesterone acetate at 2.5 mg/d; n = 8 of 19 [42%]) for a median duration of 9 (IQR, 4-13) years. Although the frequency of mild cognitive impairment diagnosis was slightly higher in the BSO group (n = 3 [13%]) compared with the control group (n = 1 [5%]; *P* = .40), the short test of mental status, global cognitive status scores, and the Beck depression and anxiety inventory scores did not differ between the groups (Table).

**Table.** Demographics, Global Cognitive Status, and Imaging Characteristics of the Study Sample^a^

Characteristic	No. (%)		*P* Value
	Bilateral Salpingo-oophorectomy (n = 23)	Control Group (n = 20)	
Demographic and clinical characteristic			
Age at oophorectomy, median (IQR), y	46 (45-48)	NA	NA
Time from oophorectomy to imaging, median (IQR), y	19 (17-22)	NA	NA
Age at imaging, median (IQR), y	65 (62-68)	63 (60-66)	.23
Length of Olmsted County residence at the time of MCSA baseline, median (IQR), y	41 (34-44)	36 (34-41)	.56
Women who reached menopause	23 (100)	19 (95)	.47
Hormone therapy with estrogen^b^	22 (96)	10 (53)	.002
Estrogen therapy, median (IQR), y^c^	10 (5-13)	10 (6-16)	.76
Oral conjugated equine estrogen^c^	15 (68)	9 (90)	.38
Hormone therapy with progestin^b^	NA	8 (42)	NA
Progestin therapy, median (IQR), y^d^	NA	9 (4-13)	NA
Oral medroxyprogesterone acetate^d^	NA	8 (100)	NA
BMI, median (IQR)	30 (27-34)	29 (25-31)	.22
Smoking^e^	5 (36)	9 (56)	.30
Education, median (IQR), y	14 (13-16)	16 (13-18)	.62
*APOE* ε4 carriers	2 (9)	4 (20)	.40
Neuropsychological evaluation scores			
Short test of mental status, median (IQR)	37 (35-37)	37 (35-38)	.20
*z* Scores, median (IQR)			
Global	1.51 (0.85-1.99)	1.59 (0.99-1.91)	.78
Memory	1.57 (0.62-2.09)	1.53 (1.00-1.89)	.73
Attention	1.43 (0.96-1.77)	1.37 (0.88-1.73)	.79
Language	1.05 (0.17-1.34)	1.01 (0.73-1.52)	.58
Visuospatial	0.73 (0.14-1.40)	1.19 (0.84-1.48)	.28
Mild cognitive impairment	3 (13)	1 (5)	.40
Beck Depression Inventory score, median (IQR)	4 (1-6)	4 (2-7)	.52
Beck Anxiety Inventory score, median (IQR)	1 (0-4)	0 (0-3)	.28
Neuroimaging biomarker measurements, median (IQR)			
White matter hyperintensity, cm^3^	5.46 (2.87-8.11)	4.13 (2.98-9.98)	.52
Hippocampal volume, score^f^	−0.32 (−0.80 to 0.29)	−0.05 (−0.29 to 0.30)	.13
Amygdala volume, cm^3^	1.74 (1.59-1.91)	2.15 (2.05-2.37)	<.001
Parahippocampal-entorhinal cortical thickness, mm	3.91 (3.64-4.00)	3.97 (3.89-4.28)	.046
Entorhinal white matter fractional anisotropy	0.19 (0.18-0.22)	0.22 (0.20-0.23)	.03
Global cortical Pittsburgh compound B standard uptake value ratio	1.29 (1.24-1.35)	1.25 (1.19-1.31)	.17
Infarctions	1 (4)	1 (5)	>.99

Abbreviations: BMI, body mass index (calculated as weight in kilograms divided by height in meters squared); IQR, interquartile range; MCSA, Mayo Clinic Study of Aging; NA, not applicable.

^a^ Wilcoxon rank sum tests, rank regression tests adjusted for total intracranial volume, and Fisher exact tests were used to compare the bilateral salpingo-oophorectomy and control groups.

^b^ In 23 women who had undergone bilateral salpingo-oophorectomy and 19 control participants who reached menopause.

^c^ In 22 women who had undergone bilateral salpingo-oophorectomy and in 10 control participants who used hormone therapy with estrogen; the typical dosage was 0.625 mg/d.

^d^ In 8 control participants who used hormone therapy with progestin; the typical dosage was 2.5 mg/d.

^e^ In 14 women who had undergone bilateral salpingo-oophorectomy and in 16 control participants with known smoking status.

^f^ Hippocampal volume was calculated as raw right plus left hippocampal volumes, adjusted for total intracranial volume. To derive the total intracranial volume–adjusted hippocampal volume, we fit a linear regression model among 133 participants with normal cognitive function aged 30 to 59 years to predict hippocampal volume from total intracranial volume.^13^

**Figure 2.**
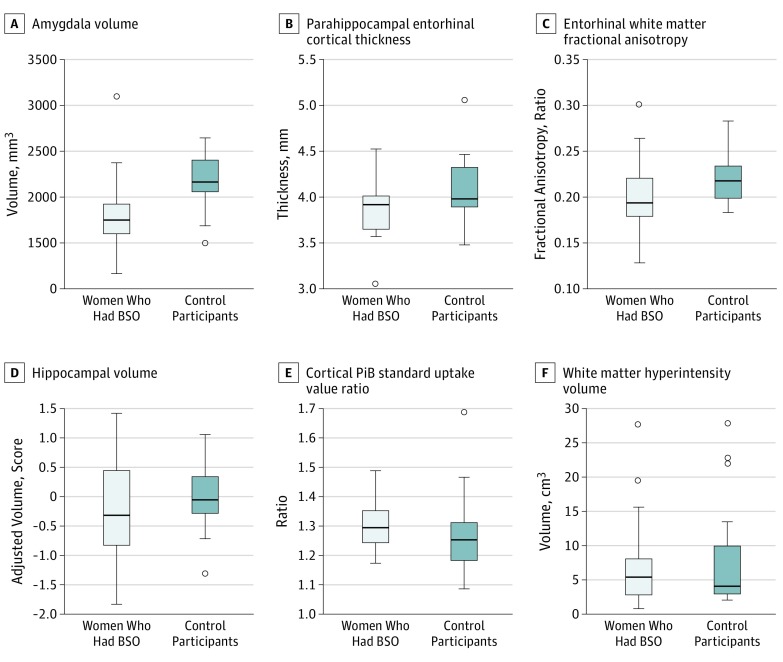
Imaging Characteristics in Women Who Underwent Bilateral Salpingo-Oophorectomy (BSO) vs Control Participants Box plots show median and interquartile ranges. Amygdala volume was smaller and parahippocampal-entorhinal cortex was thinner on structural magnetic resonance imaging, and entorhinal white matter fractional anisotrophy was lower on diffusion tensor imaging in women who had undergone BSO than in control participants. Hippocampal volume was calculated as raw right plus left hippocampal volumes, adjusted for total intracranial volume. To derive the total intracranial volume–adjusted hippocampal volume, we fit a linear regression model among 133 participants with normal cognitive function aged 30 to 59 years to predict hippocampal volume from total intracranial volume.^13^ There were no statistically significant differences in hippocampal volume, cortical Pittsburgh compound B (PiB) standard uptake value ratio, or white matter hyperintensity volume between women who had undergone BSO and control participants.

On structural MRI, median (IQR) amygdala volume was smaller (BSO group, 1.74 [1.59-1.91] cm^3^; control group: 2.15 [2.05-2.37] cm^3^; *P* < .001), median (IQR) parahippocampal-entorhinal cortex was thinner (BSO group: 3.91 [3.64-4.00] mm; control group: 3.97 [3.89-4.28] mm; *P* = .046), and the entorhinal white matter fractional anisotropy on diffusion tensor imaging was lower (BSO group: 0.19 [0.18-0.22]; control group: 0.22 [0.20-0.23]; *P* = .03) in the BSO group compared with the control group (Table). Smaller hippocampal volume on MRI and higher cortical PiB standard uptake value ratio on PiB-PET scan were observed in the BSO group compared with the control group, but these results did not reach statistical significance (Figure 2). The results did not change noticeably in sensitivity analyses that added 4 women who had an MRI but not a PiB-PET scan, and in sensitivity analyses that removed 4 women who had mild cognitive impairment at the time of imaging testing (data not shown).

White matter hyperintensity volume and the frequency of infarctions did not differ between the groups. Imaging biomarkers were not associated with the global cognitive status score, and Beck Anxiety Inventory and Beck Depression Inventory scores after correcting for multiple comparisons using false-discovery rate.

## Discussion

In this study, women who underwent BSO before age 50 years and before reaching natural menopause had smaller amygdala volumes, thinner parahippocampal-entorhinal cortices, and lower entorhinal white matter fractional anisotropy values compared with control-participant women.

There is an increased risk of cognitive impairment or dementia^1^ and presence of AD pathology^4^ in women who undergo BSO before menopause. However, imaging biomarkers associated with AD pathophysiology that precede cognitive impairment have not been studied in these women. Results of the present study suggest that women who underwent early BSO have biomarker abnormalities associated with neurodegeneration in the medial temporal lobe. In particular, the entorhinal cortex, which is one of the regions involved with neurofibrillary tangle pathology during the preclinical stages of AD, as well as primary age-associated taupathy.^14^ In addition, a thinner entorhinal cortex and lower fractional anisotropy on diffusion tensor imaging in the BSO group suggest a disruption in the entorhinal white matter microstructure that includes the perforant pathway carrying the connections between the entorhinal cortex and the hippocampus. Although not statistically significant in this small explorative sample, a difference in hippocampal volumes and cortical PiB uptake in the BSO group compared with the control group suggests the occurrence of early biomarker changes associated with AD pathophysiology.

Premenopausal oophorectomy–induced estrogen deficiency is thought to be the primary cause of the increased risk of cognitive impairment or dementia in women with BSO before the onset of menopause.^1^ Furthermore, AD biomarker abnormalities have been observed more frequently in women undergoing menopause compared with premenopausal women, after controlling for age.^15^ In our study, 96% of the women with BSO were treated with estrogen (primarily oral conjugated equine estrogen), for a median of 10 years after BSO; however, this type and duration of hormonal treatment after BSO does not seem to be sufficient to prevent structural changes in the medial temporal lobe later in life. Further research into the type of estrogen treatments used, route of administration, dosing, and influence of the other ovarian hormones and hormones of the pituitary-ovarian axis is needed.

### Limitations

Lower hippocampal volume and higher cortical β-amyloid accumulation observed in the BSO group compared with the control group may have failed to reach statistical significance because of the small sample size. Because structural imaging biomarkers of medial temporal lobe neurodegeneration are associated with cognitive impairment and dementia later in life,^16^ findings of the current study support the association between BSO in premenopausal women and an increased risk of cognitive decline and dementia.^1,4^

## Conclusions

Abrupt hormonal changes because of BSO in premenopausal women may lead to medial temporal lobe structural abnormalities later in life. Because alterations in structural imaging biomarkers of neurodegeneration in the medial temporal lobe precede clinical symptoms of dementia, enlargement and longitudinal follow-up of this cohort is needed.
